# Impact of Maternal Diet on Human Milk Composition Among Lactating Women in Latvia

**DOI:** 10.3390/medicina55050173

**Published:** 2019-05-20

**Authors:** Līva Aumeistere, Inga Ciproviča, Dace Zavadska, Juris Andersons, Viktors Volkovs, Kristīne Ceļmalniece

**Affiliations:** 1 Faculty of Food Technology, Latvia University of Life Sciences and Technologies, Rīgas iela 22, LV-3004 Jelgava, Latvia; inga.ciprovica@llu.lv; 2 Institute of Food Safety, Animal Health and Environment BIOR, Lejupes iela 3, LV-1076 Riga, Latvia; juris.andersons@bior.lv (J.A.); viktors.volkovs@bior.lv (V.V.); kristine.celmalniece@bior.lv (K.C.); 3 Department of Pediatrics, Rīga Stradiņš University, Vienības gatve 45, LV-1004 Riga, Latvia; dace.zavadska@rsu.lv

**Keywords:** human milk, lactation, fat, protein, lactose, fatty acids, diet

## Abstract

*Background and objectives*: Many studies indicate that the maternal diet is an important factor affecting human milk composition. Human milk composition among lactating women in Latvia, as well as the maternal diet during lactation, has not been sufficiently studied. The aim of this research was to assess dietary habits and macronutrient intake among lactating women in Latvia and to examine the effect of diet on human milk composition. *Materials and Methods*: Research was conducted between November 2016 and December 2017. Mature human milk samples (*n* = 61) along with a 72h food diary, a food frequency questionnaire (FFQ), and a questionnaire about maternal and infant characteristics were obtained from voluntary women who were recruited via an invitation published in a social media member group for nursing mothers. Fat content in human milk was determined by LVS ISO 2446:2008, protein content was determined by LVS EN ISO 8968-1:2014, lactose was determined by ISO 22662:2007, and the fatty acid profile was analyzed using gas chromatography. Dietary data were evaluated using the Finnish food composition database Fineli, release 19 (3 March 2018). *Results*: Median values for fat, protein, and lactose in mature human milk were 4.40%, 1.08%, and 6.52%, respectively. Predominant fatty acids in human milk were oleic acid (C18:1 n9*c*), palmitic acid (C16:0), and linoleic acid (C18:2 n6*c*) at 34.60%, 24.00%, and 11.00% of total fatty acids, respectively. The *trans* elaidic acid (C18:1 n9*t*) level was <0.10% in all human milk samples. Significant, positive associations (*p* < 0.05) were found between maternal dietary intake of linoleic, α-linolenic, docosahexaenoic, total *cis*-monounsaturated, total *cis*-polyunsaturated, and total n-6 and n-3 polyunsaturated fatty acids, the ratio of n-6/n-3, and the level of these fatty acids in human milk. Total energy and carbohydrate intake among participants were lower, but total fat, saturated fat, and sugar intake were higher than recommended. Protein, linoleic acid, and α-linolenic acid intake were adequate, but docosahexaenoic acid intake was noticeably lower than recommended. Women should be supported with information regarding their nutritional needs during lactation and the possible impact of diet on human milk composition. *Conclusion*: Macronutrient (fat, protein, and lactose) content in human milk is not affected by maternal diet. Conversely, the human milk fatty acid profile is affected by the immediate diet consumed by the mother. Habitual dietary habits can also impact the fatty acid profile of human milk.

## 1. Introduction

Adequate nutrient intake during the first year of life is critical for an infant’s proper growth and development. In the first half of the year, human milk is an essential source of nutrients. After, women are encouraged to continue breastfeeding jointly with suitable complementary food [1]. Human milk contains many components including proteins, carbohydrates, and lipids [2].

Proteins in human milk are divided into three groups—caseins, whey proteins (α-lactalbumin, lactoferrin, and secretory immunoglobulin A), and milk fat globule membrane proteins [3]. Lactose composes ~98% of total carbohydrates in human milk and serves as a source of energy for the infant [2].

Approximately half of total energy intake in human milk is derived from fat, mostly in the form of triacylglycerols (~98%) [2]. Human milk triacylglycerols comprise medium-chain (C10–C14) and long-chain fatty acids (C16–C24) [2]. Fatty acids containing up to 14 carbon atoms are de novo synthesized in the mammary glands. Longer chain fatty acids (≥C16) are transferred from endogenous stores (maternal adipose tissue), released from the liver, or accumulated from the current maternal diet [2]. Fatty acids from dietary intake are promptly transferred (within six hours) to human milk, and the level of all fatty acids in human milk can stay significantly elevated for up to three days from initial ingestion [2,4]. Docosahexaenoic acid (22:6 n-3) is the main n-3 long chain fatty acid, but arachidonic acid (20:4 n-6) is the main n-6 long chain fatty acid found in cell membrane phospholipids, especially in the brain and retina [5,6]. In the human body, docosahexaenoic acid can be synthesized from α-linolenic acid (18:3 n-3), and arachidonic acid is synthesized from linoleic acid (18:2 n-6*c*). Human body cells lack enzymes (Δ12 and Δ15 desaturase); therefore, linoleic acid (18:2 n-6*c*) and α-linolenic acid (18:3 n-3) are essential and must be consumed from the diet [6]. Although both n-6 and n-3 fatty acids are vital, they have opposite functions in the human body. n-6 fatty acids are precursors for eicosanoids with inflammatory effects, and n-3 fatty acids are precursors for eicosanoids displaying anti-inflammatory effects [7]. Both α-linolenic acid and linoleic acid mutually compete for the converting enzyme Δ6 desaturase in the synthesis of long-chain fatty acids (docosahexaenoic or arachidonic acid) [7]. Excess intake of n-6 fatty acids can impact the bioavailability of n-3 fatty acids [7,8]. A high n-6/n-3 ratio (≥15/1) promotes inflammation and leads to many chronic diseases (cardiovascular, inflammatory, autoimmune, etc.) [7].

Sufficient long-chain polyunsaturated fatty acid intake during the first two years of life is critical for the growth and proper development of the central nervous system [5]. Although docosahexaenoic acid and arachidonic acid are nonessential fatty acids, infants have a limited capacity to endogenously convert fatty acids. Approximately 28% of plasma α-linolenic acid is converted to docosahexaenoic acid [9]. Therefore, during infancy, human milk is a vital source of both essential and long-chain n-6 and n-3 polyunsaturated fatty acids [8]. Mothers should consume a well-balanced diet to ensure sufficient polyunsaturated fatty acid transfer via human milk [10]. According to recommendations, women during lactation should consume at least 200 mg of docosahexaenoic acid per day, but total n-3 polyunsaturated fatty acid intake should be at least 1% of total energy intake [10].

*Trans* fatty acid synthesis is limited in the human body; therefore, the main source for these fatty acids in human milk is the maternal diet [11]. The main *trans* isomers in partially hydrogenated vegetable oils are elaidic acid (C18:1 n-9*t*) and linolelaidic acid (C18:2 n-6*t*), and in ruminant milk the main *trans* fat is vaccenic acid (C18:1 n-11*t*) [11]. Intake of industrially produced *trans* fatty acids (predominantly elaidic acid) via human milk resulted in growth delay in mice [12]. Human studies show that *trans* fatty acids may also impact n-6 and n-3 polyunsaturated fatty acid metabolism in infants [13]. Taking into account the possible negative effects of *trans* fatty acids on health, maternal intake of these fatty acids should be kept as low as possible [10]. However, it should be also taken into account that some *trans* fatty acids may have beneficial effects. Conjugated linoleic acid isomers display anticarcinogen attributes [14]. A small portion (<10%) of rumenic acid (C18:2 n-9*c*, n11*t*) can be synthesized from vaccenic acid through endogenous desaturation in lactating women [15].

Breastfeeding rates in Latvia are increasing every year [16]. Yet, there is still an insufficient amount of information regarding human milk composition among lactating women from Latvia [17]. Many studies indicate that maternal diet can influence human milk composition, especially the fatty acid profile [18,19]; therefore, the aim of this research was to assess dietary habits and macronutrient intake among lactating women in Latvia and to examine the effect of diet on human milk composition.

## 2. Materials and Methods

Research was conducted between November 2016 and December 2017. Approval from Rīga Stradiņš University Ethics Committee was acquired prior to the start of research (No. 4/28.7.2016.). Women were recruited via an invitation published in a social media member group for nursing mothers, and all of them voluntarily signed informed consent. Participants were instructed to complete a food diary for 72 h and to collect a pooled human milk sample (~60 mL) in the next 24 h. Mothers were allowed an ad libitum diet and chose the most convenient method for milk expression (by hand, using a breast pump, or a combination of both methods). In order to make quantification of food easier, a food atlas [20] and size measures (spoons, etc.) were used. To ensure that mature human milk was collected, only women that were at least one month postpartum were included in the study (*n* = 61). Only hindmilk was collected, and the pooled sample had to include milk from morning, midday, and evening feedings, yet sampling frequency and time were not specified. Samples were kept frozen until analysis and thawed only once.

Fat content in human milk was determined by LVS ISO 2446:2008, protein content was determined by LVS EN ISO 8968-1:2014 (conversion factor of 6.25 was used), lactose was determined by ISO 22662:2007, and the fatty acid profile of human milk was analyzed using gas chromatography (for a more detailed description, see Aumeistere et al., 2018 [21]).

Dietary software with national food data from Latvia lacks information regarding fatty acid composition; therefore, daily total energy and macronutrient intake from the food diary were computed using the Finnish food composition database Fineli, database release 19, (3 March 2018), which was freely accessible online [22]. Calculations from the Fineli food composition database were exported to MS Excel 2013. Data about dietary supplements (if participants had indicated) were taken from the manufacturer’s website. Participants also completed a food frequency questionnaire (FFQ) about the consumption frequency of different food products one month prior to the study as well as a questionnaire about maternal and infant characteristics (Table 1).

Statistical processing of data was done using IBM SPSS Statistics, version 22.0. Related-samples Friedman’s two-way analysis of variance by ranks was used to evaluate the distribution among energy and nutrient intake from the diary. Spearman correlation, partial correlation, and Mann–Whitney U tests were used to evaluate if human milk composition was affected by maternal and infant characteristics or maternal diet (α = 0.05).

## 3. Results

### 3.1. Macronutrients in Human Milk

Median values and ranges for fat, protein, and lactose content in mature human milk are compiled in Table 2. Fat content in human milk was significantly higher for mothers practicing partial breastfeeding (fat content 4.83%) than mothers who exclusively breastfed their infants (fat content 4.10%) (*p* = 0.033). Protein content in human milk negatively correlated with infant age (*r* = −0.413, *p* = 0.001).

Median values and ranges for fatty acid levels in mature human milk are summarized in Table 3. The predominant fatty acids in human milk were oleic acid (C18:1 n9*c*), palmitic acid (C16:0), and linoleic acid (C18:2 n6*c*) at 34.60%, 24.00%, and 11.00% of total fatty acids, respectively. Feeding patterns or any other maternal and infant characteristics did not influence fatty acid composition of human milk (*p* > 0.05).

### 3.2. Dietary Habits and Macronutrient Intake Among Lactating Women from Latvia

The FFQ (Table 4) revealed that most of the major food groups were not included in the everyday diet among participants. The majority of women noted seldom consumption of grains, cereals, and cereal products. Milk and dairy products were mostly consumed twice a week. Eggs, meat, and meat products were mostly consumed once a week. The majority of participants avoided or seldom consumed fish and seafood. Also, fruits and vegetables were consumed only once a week by the majority of participants.

After adjusting for potential confounders (maternal age, infant age and sex, parity, and feeding pattern), significant positive associations were found between fish consumption frequency and docosahexaenoic acid levels in human milk (*r* = 0.273, *p* = 0.042) as well as nut and seed consumption frequency and linoleic acid levels in human milk (*r* = 0.281, *p* = 0.036). Frequency of milk and dairy product consumption was negatively correlated with polyunsaturated fatty acid (*r* = −0.492, *p* = 0.000),both n-3 (*r* = −0.398, *p* = 0.002) and n-6 polyunsaturated fatty acids (*r* = −0.426, *p* = 0.001), linoleic acid (*r* = −0.413, *p* = 0.002), and α-linolenic acid (*r* = −0.280, *p* = 0.036) levels in human milk. Conversely, milk and dairy consumption was positively correlated with saturated fatty acid (*r* = 0.485, *p* = 0.000) levels in human milk. Egg, meat, and meat product consumption frequencies were negatively correlated with polyunsaturated fatty acid (*r* = –0.366, *p* = 0.005), n-6 polyunsaturated fatty acid (*r* = −0.330, *p* = 0.013), linoleic acid (*r* = −0.337, *p* = 0.011), and α-linolenic acid (*r* = −0.323, *p* = 0.015) levels in human milk, but it was positively correlated with *trans* fatty acid (*r* = 0.291, *p* = 0.029) levels in human milk. Lower *trans* fatty acid levels in human milk were found in participants who more frequently consumed grains, cereals, and cereal products (*r* = −0.291, *p* = 0.029), vegetables and legumes (*r* = −0.287, *p* = 0.032), and nuts and seeds (*r* = −0.281, *p* = 0.036).

Data analysis of the diaries revealed that the distributions among energy and nutrient intake in all three consecutive days were the same (*p* > 0.05); therefore, median values from all three days were further used in the calculations (Table 5). The median percentage distribution of total energy intake among participants was ~ 41% from carbohydrates, ~16% from protein, and ~40% from fat. Saturated fatty acid intake constituted ~14%, monounsaturated fatty acid intake ~14%, polyunsaturated fatty acid intake ~7%, n-6 fatty acid intake ~5%, n-3 fatty acid intake ~1%, linoleic acid intake ~5%, and α-linolenic acid intake ~1% of total energy intake. Total sugar intake was ~21% of total energy intake.

Grain, cereal, and cereal product consumption frequency was associated with lower total fat intake among the participants (*r* = –0.292, *p* = 0.029). Participants who consumed eggs, meat, and meat products more frequently, on average, consumed more protein (*r* = 0.440, *p* = 0.001) and saturated fatty acids (*r* = 0.282, *p* = 0.035) but less fiber (*r* = –0.406, *p* = 0.002). Participants with higher fish intake consumed more eicosapentaenoic (*r* = 0.449, *p* = 0.001) and docosahexaenoic acids (*r* = 0.478, *p* = 0.000) but less α-linolenic acid (*r* = –0.293, *p* = 0.028). Vegetable and legume consumption frequency was associated with higher fiber (*r* = 0.326, *p* = 0.014) but lower *trans* fatty acid intake (*r* = –0.370, *p* = 0.005). Fruit and berry consumption frequency was associated with higher carbohydrate (*r* = 0.277, *p* = 0.039) and fiber (*r* = 0.423, *p* = 0.001) intake. Participants with higher butter, margarine, and fat spread consumption consumed less fiber (*r* = –0.344, *p* = 0.009) but more saturated fatty acids (*r* = 0.337, *p* = 0.011) and *trans* fatty acids (*r* = 0.301, *p* = 0.024). Participants with a higher sweet and bakery product consumption frequency consumed more saturated fatty acids (*r* = 0.472, *p* = 0.000) and *trans* fatty acids (*r* = 0.377, *p* = 0.004) but less of n-3 polyunsaturated fatty acids (*r* = –0.285, *p* = 0.033).

After adjusting for potential confounders (maternal age, infant age and sex, parity, and feeding pattern), there were still significant positive associations between fatty acid levels and dietary intake of linoleic acid, α-linolenic acid, docosahexaenoic acid, total *cis*-monounsaturated, total *cis*-polyunsaturated, total n-6 and n-3 polyunsaturated fatty acids, and ratio of n-6/n-3 in human milk (Table 6).

## 4. Discussion

Human milk composition among lactating women in Latvia, as well as maternal diet during lactation, has not been sufficiently studied before. Our median fat value (4.40%) was higher compared to data reported by Ballard and Morrow (3.2–3.6%) [25]. This could be due to fact that we analyzed hindmilk, which is fattier than foremilk. Fat is the most variable nutrient in human milk [2], also fat content in human milk among participants in our study was quite variable (1.00–7.70%). Obtained results could be explained by the sampling procedure used in our study. To take into account possible diurnal variations, we analyzed pooled 24 h human milk samples and asked mothers to obtain hindmilk from morning, midday, and evening feedings. Yet, sampling frequency and time were not specified. Currently, there is no “gold standard” for human milk sampling, and different approaches have been used among researchers [26]. Similar to us, Mitoulas et al. [27] analyzed 24 h human milk samples and noted that fat content in human milk can vary significantly among women. We observed that fat content in human milk was significantly higher for mothers practicing partial breastfeeding than mothers who exclusively breastfed their infants. This could be explained by the fact that variation in fat content was affected by the breastfeeding frequency. With lesser breastfeeding frequency (six to nine times per day), breasts are more drained from milk during each feed, resulting in lower fat content in foremilk (~4.3%) and increased fat content in hindmilk (~10.7%) compared to more frequent breastfeeding (14 to 18 times per day, ~4.8% of fat for foremilk and ~8.2% of fat for hindmilk) [28].

Median protein content in our study was 1.08% and was within the range reported by Ballard and Morrow (0.9–1.2%) [25]. We observed that protein content in human milk was negatively correlated with the infant’s age. A tendency that protein content decreases as lactation progresses has also been observed by Saarela et al. [29].

Our obtained median content for lactose (6.52%) was lower compared to values reported by Grote et al. (7.24–8.03%) [30]. According to our data and other studies [29,31], lactose content in mature human milk seems to remain stable and is not affected by maternal or infant characteristics.

There were three dominating fatty acids in the human milk samples: palmitic acid (11%), oleic acid (34.60%), and linoleic acid (24.00%). This coincides with data from studies all around the world [32,33,34,35,36,37,38,39,40,41]. Dominance of unsaturated fatty acids over saturated helps to retain human milk fluidity and lipid digestion for breastfed infants [37].

Medium-chain fatty acids comprise around 15% of the fatty acids of mature human milk [2]. A slightly lower value for the medium-chain fatty acid level was observed in our study (12.40%). Although some studies indicated [42] that higher carbohydrate intake was associated with higher medium-chain fatty acid levels in human milk, this relation was not found in our study (*r* = 0.166, *p* = 0.223).

Both sufficient intake of docosahexaenoic acid and arachidonic acid, and their precursors α-linolenic and linoleic acid, are vital during infancy [5,6,43]. Linoleic acid (11%) and α-linolenic acid (1%) levels in human milk in our study were similar to values obtained in other studies across Europe —11% and 1.1%, respectively [44]. Also, median docosahexaenoic acid levels in human milk (0.30%) in our study corresponded to the mean worldwide level of docosahexaenoic acid (0.32%) [45]. Arachidonic acid (0.30%) levels among participants were lower than the worldwide level of this fatty acid—0.47% [45]. It is well documented [36,45] that docosahexaenoic acid levels in human milk are affected by maternal intake of this fatty acid, which is in agreement with our results. However, it seems that arachidonic levels in human milk are less affected by maternal intake [45] but preferentially may come from endogenous stores (adipose tissue) [2]. Arachidonic acid is formed from linoleic acid and is vital for normal growth and development of the brain [43]. Arachidonic acid is also needed as a precursor for the synthesis of eicosanoids; therefore, it is important for growth [43]. Further research should be done to evaluate why lower arachidonic levels in human milk were observed in our study.

Industrially produced *trans* fatty acids consumed by a lactating woman are incorporated into adipose tissues and then passed to an infant via human milk [13]. The main *trans* isomer in partially hydrogenated vegetable oils (elaidic acid) is associated with growth delay in mice [11,12]. Elaidic acid levels in human milk in our study (<0.10% in all samples) were the lowest obtained values compared to data from other countries [31,46,47,48,49]. Dietary intake of *trans* fatty acids was also low among participants (0.00–2.13g). A significant association between *trans* fatty acid intake and *trans* fatty acid levels in human milk was not found (*p* = 0.418). Unfortunately, dietary software of national food data from Latvia lacks information regarding *trans* fatty acid content in different foodstuffs; therefore, we used the Finish food database Fineli [22] to evaluate food diary records among the participants. On 20 May 2016, Cabinet Regulation of Republic of Latvia No. 301 “Regulations regarding the maximum permitted amount of *trans* fatty acids in food products” entered in force [50]. The transition period was until 1 June 2018. Many producers may have implemented the new requirements before the deadline, and this could explain the obtained low levels of elaidic acid in human milk.

Median energy intake among participants (2044 kcal) was lower than recommended levels for lactating women according to guidelines developed by the Ministry of Health of the Republic of Latvia [23]. Fat intake was higher than recommended (~40% of total energy intake instead of the recommended 25 to 30%) [23]. Protein intake was within range (10 to 20% of total energy intake), but carbohydrate intake was lower than recommended (41% of total energy intake instead of the recommended 45 to 60%) [23]. Saturated fat intake was higher than recommended (14 instead of 10% of total energy intake) [23]. Sugar intake among participants was twice as high as recommended (21% instead of ≤10% of total energy intake) [23]. Guidelines developed by the Ministry of Health of the Republic of Latvia [23] do not specify fatty acid intake, but according to the European Food Safety Authority guidelines [24], linoleic acid intake (4.0% of total energy intake) and α-linolenic acid intake (0.5% of total energy intake) among our participants were adequate. Median daily docosahexaenoic acid intake was lower than that recommended by the Nordic Nutrition Recommendations [10] (85.65 mg instead of 200 mg). Fish consumption frequency (a good source of docosahexaenoic acid [22]) was also low. Yet, a median docosahexaenoic acid level in human milk (0.30%) fit the target value set by Jackson and Harris [51]. A regression analysis equation was developed by Brenna and Lapillonne [52] to calculate docosahexaenoic acid levels in human milk according to maternal docosahexaenoic acid intake:

Docosahexaenoic acid level in human milk (%) = 0.72 × maternal docosahexaenoic acid intake (grams per day) + 0.20.(1)

With our median docosahexaenoic acid intake among the participants, levels of this fatty acid in human milk should be ~0.26%. In fact, we obtained a higher median docosahexaenoic acid level in human milk (0.30%). The difference could be due to the large variance in docosahexaenoic acid intake among the participants (0.00–4664.61 mg).

European Food Safety Authority [53] states that currently there are insufficient data to set a value for the n-6/n-3 ratio, but it should be as low as possible in order to reduce the risk of many chronic diseases (cardiovascular, inflammatory, autoimmune, etc.) [7]. The ratio of n-6/n-3 in human milk samples was ~7, which was lower than found in human milk samples among women from Croatia (~11), Greece (ratio of ~26), Spain (ratio of ~13), and Turkey (~32%) [18,32,54,55].

## 5. Conclusions

Macronutrient (fat, protein, and lactose) content in human milk is not affected by maternal diet. Conversely, the human milk fatty acid profile is affected by the immediate diet consumed by the mother—significant positive correlations were found among linoleic, α-linolenic, docosahexaenoic, monounsaturated, and polyunsaturated fatty acids, n-6/n-3 ratio intake, and relevant fatty acid levels in human milk. Habitual dietary habits can also impact the fatty acid profile of human milk. Total energy and carbohydrate intake among participants were lower, but total fat, saturated fat, and sugar intake were higher than recommended. Protein, linoleic acid, and α-linolenic acid intake were adequate, but docosahexaenoic acid intake was noticeably lower than recommended. Women should be supported with information regarding their nutritional needs during lactation and the possible impact of diet on human milk composition.

## Figures and Tables

**Table 1:** Maternal and infant characteristics (*n* = 61).

Parameter	Median Value	Range
**Maternal Characteristics**		
Maternal age (years)	31	23–39
Parity (*n*)	2	1–4
Feeding pattern	37—exclusive breastfeeding 24—partial breastfeeding	
Milk expression manner	16—by hand 36—using a breast pump 9—breast pump + by hand	
**Infant and Toddler Characteristics**		
Age (months)	4.0	1.50–21.0
Sex	27 girls, 34 boys	

**Table 2:** Fat, protein, and lactose content in mature human milk (*n* = 61).

Macronutrient	Median Value	Range
Fat, %	4.40	1.00–7.70
Protein, %	1.08	0.75–1.82
Lactose, %	6.52	3.29–7.30

**Table 3:** Fatty acid profile of mature human milk (*n* = 61).

Fatty Acids	Median Value, % of Total Fatty Acids	Range, % of Total Fatty Acids
Fatty acids, total saturated	45.70	27.20–60.80
Fatty acids, medium chain (10:0–14:0)	12.40	6.20–24.50
Fatty acids, total *cis*-monounsaturated	37.80	28.50–44.10
Fatty acids, total *cis*-polyunsaturated	13.60	7.30–31.70
Fatty acids, total n-6 *cis*-polyunsaturated, of which:	12.10	5.60–27.30
Linoleic acid (18:2 n-6*c*)	11.00	5.00–26.50
Arachidonic acid (20:4 n-6)	0.30	0.10–0.50
Fatty acids, total n-3 *cis*-polyunsaturated, of which:	1.60	0.90–10.30
α-Linolenic acid (18:3 n-3)	1.00	0.50–9.60
Eicosapentaenoic acid (20:5 n-3)	0.10	<0.10–2.70
Docosahexaenoic acid (22:6 n-3)	0.30	0.10–4.30
Ratio n-6/n-3	7.00	1.86–22.70
Fatty acids, total *trans*, of which:	1.40	<0.10–2.80
Elaidic acid (C18:1 n-9*t*)	<0.10	<0.10
Vaccenic acid (C18:1 n-11*t*)	1.20	<0.10–1.90
Linolelaidic acid (C18:2 n-6*t*)	0.20	<0.10–1.50

**Table 4:** Average intake frequency (%) of food products among participants one month prior to the study (*n* = 61).

Food Products	Never	Seldom	Once a Week	Twice a Week	More Than Twice a Week but not Every Day	Every Day
Grains, cereals, and cereal products	3%	67%	30%	0%	0%	0%
Eggs, meat, and meat products	8%	15%	52%	23%	2%	0%
Fish	20%	33%	33%	6%	6%	2%
Seafood	39%	52%	7%	2%	0%	0%
Milk and dairy products	16%	3%	18%	46%	16%	0%
Vegetables and legumes	0%	12%	66%	23%	0%	0%
Fruits and berries	2%	30%	49%	18%	2%	0%
Nuts and seeds	5%	30%	26%	8%	20%	12%
Butter, margarine, and fat spreads	26%	39%	31%	3%	0%	0%
Vegetable oils	2%	3%	5%	12%	46%	33%
Condiments	16%	57%	25%	2%	0%	0%
Sweets and bakery products	11%	44%	41%	3%	0%	0%
Salty snacks and fast food	34%	54%	11%	0%	0%	0%
Sugared, carbonated drinks	59%	34%	5%	0%	2%	0%
Caffeine-containing drinks	8%	13%	25%	28%	20%	7%
Herbal teas	3%	5%	8%	20%	25%	39%
Alcohol	59%	31%	8%	2%	0%	0%

**Table 5:** Daily energy and macronutrient intake of lactating women (*n* = 61).

Parameter	Median Value	Range	Dietary Recommendations
Energy (kcal)	2044	992–3844	1960–2510 kcal (18–30 y) + 500–600 kcal ^1^ 1840–2360 kcal (>31 y) + 500–600 kcal ^1^
Carbohydrates (g)	209.28	75.51–410.21	45–60% of total energy intake ^1^
Fiber, total (g)	21.82	6.79–100.42	25–35 g
Sugars (g), of which:	103.48	28.24–326.93	Not more than 10% of total energy intake ^1^
Glucose (g)	17.64	1.21–71.13	-
Fructose (g)	17.83	1.11–78.16	-
Sucrose (g)	48.36	1.06–244.14	-
Maltose (g)	0.29	0.00–3.26	-
Galactose (g)	0.00	0.00–12.00	-
Lactose (g)	6.62	0.00–43.08	-
Protein, total (g)	80.42	30.45–190.47	10–20% of total energy intake ^1^
Fat, total (g)	87.29	25.32–304.01	25–30% of total energy intake ^1^
Fatty acids, total saturated (g)	31.29	8.02–90.79	Not more than 10% of total energy intake ^1^ As low as possible ^2^
Fatty acids, total *cis*-monounsaturated (g)	31.30	7.66–90.07	10–20% of total energy intake ^3^
Fatty acids, total *cis*-polyunsaturated (g)	13.92	1.27–66.76	5–10% of total energy intake ^3^
Fatty acids, total n-6 *cis*-polyunsaturated (g), of which:	10.43	0.81–47.75	Together with n-3 polyunsaturated fatty acids at least 5% of total energy intake ^3^
Linoleic acid (18:2 n-6*c*) (mg)	10610.53	1096.62–47821.29	4% of total energy intake ^2^
Fatty acids, total n-3 *cis*-polyunsaturated (g), of which:	2.10	0.00–31.12	At least 1% of total energy intake ^3^
α-linolenic acid (18:3 n-3) (mg)	1903.85	261.83–31128.36	At least 0.5% of total energy intake ^2,3^
Eicosapentaenoic acid (20:5 n-3) (mg)	21.25	0.00–2189.45	250 mg of eicosapentaenoic acid + docosahexaenoic acid ^2^
Docosahexaenoic acid (22:6 n-3) (mg)	85.65	0.00–4664.61	250 mg of eicosapentaenoic acid + docosahexaenoic acid + 100 to 200 mg of docosahexaenoic acid ^2^ 200 mg ^3^
Ratio n-6/n-3	4.71	0.00–63.79	-
Fatty acids, total *trans* (g)	0.62	0.00–3.00	As low as possible ^2,3^

^1^: [23], ^2^: [24], ^3^: [10].

**Table 6:** Partial correlation coefficients (*r*) among human milk components and corresponding component intake by participating women (*n* = 61).

Human Milk vs Maternal Diet	*r*	*p*-Value
Fat	0.052	0.705
Protein	0.009	0.946
Lactose	0.213	0.115
Fatty acids, total saturated	0.253	0.060
Fatty acids, total *cis*-monounsaturated	0.354	0.007
Fatty acids, total *cis*-polyunsaturated	0.492	0.000
Fatty acids, total n-6 *cis*-polyunsaturated	0.455	0.000
Linoleic acid (18:2 n-6*c*)	0.458	0.000
Fatty acids, total n-3 *cis* polyunsaturated	0.723	0.000
α-linolenic acid (18:3 n-3)	0.869	0.000
Eicosapentaenoic acid (20:5 n-3)	0.093	0.495
Docosahexaenoic acid (22:6 n-3)	0.671	0.000
Ratio n-6/n-3	0.491	0.000
Fatty acids, total *trans*	0.110	0.418
